# Antimicrobial Resistance and Its Spread Is a Global Threat

**DOI:** 10.3390/antibiotics11081082

**Published:** 2022-08-09

**Authors:** Mohammed M. Aljeldah

**Affiliations:** Department of Clinical Laboratory Sciences, College of Applied Medical Sciences, University of Hafr Al Batin, Hafar al-Batin 31991, Saudi Arabia; mmaljeldah@uhb.edu.sa

**Keywords:** antimicrobial resistance, Next Generation Sequencing, antibiotics, molecular diagnosis

## Abstract

Antimicrobial resistance (AMR) is a challenge to human wellbeing the world over and is one of the more serious public health concerns. AMR has the potential to emerge as a serious healthcare threat if left unchecked, and could put into motion another pandemic. This establishes the need for the establishment of global health solutions around AMR, taking into account microdata from different parts of the world. The positive influences in this regard could be establishing conducive social norms, charting individual and group behavior practices that favor global human health, and lastly, increasing collective awareness around the need for such action. Apart from being an emerging threat in the clinical space, AMR also increases treatment complexity, posing a real challenge to the existing guidelines around the management of antibiotic resistance. The attribute of resistance development has been linked to many genetic elements, some of which have complex transmission pathways between microbes. Beyond this, new mechanisms underlying the development of AMR are being discovered, making this field an important aspect of medical microbiology. Apart from the genetic aspects of AMR, other practices, including misdiagnosis, exposure to broad-spectrum antibiotics, and lack of rapid diagnosis, add to the creation of resistance. However, upgrades and innovations in DNA sequencing technologies with bioinformatics have revolutionized the diagnostic industry, aiding the real-time detection of causes of AMR and its elements, which are important to delineating control and prevention approaches to fight the threat.

## 1. Introduction

In the year 1928, Alexander Fleming discovered penicillin, which killed the staphylococcal bacteria he was examining. It soon became the key antibiotic for the treatment of key diseases caused by Gram-positive pathogens [1]. Penicillin marked the first natural antibiotic to be discovered, though salvarsan was first deployed in the year 1910. The discovery of antibiotics has been regarded to be the bedrock of many of the greatest medical advances of the 20th century [2]. The era of antibiotic discovery made the therapeutic and surgical aspects of clinical medicine relatively safer, including procedures for childbirth, organ transplants, and cancer treatment [3]. Apart from antibiotics, antivirals have also transformed the clinical management aspects of viral diseases, including the recent coronavirus pandemic of 2019. However, one of the greatest attributes of antivirals is the ability to make the human immunodeficiency virus (HIV) manageable.

Microbial pathogens continue to evolve and develop resistance to emerging and traditional treatment methodologies; and this, combined with the extreme decline in antibiotic research has only increased the magnitude of AMR and its impacts on global healthcare costs and outcomes. The rampant upswing in use of antibiotics with over-the-counter options has only fueled AMR. This also adds to the risk of re-emergence of many diseases; the emergence of XDR-TB, extensively drug-resistant tuberculosis, is an example worth citing.

**Figure 1:**
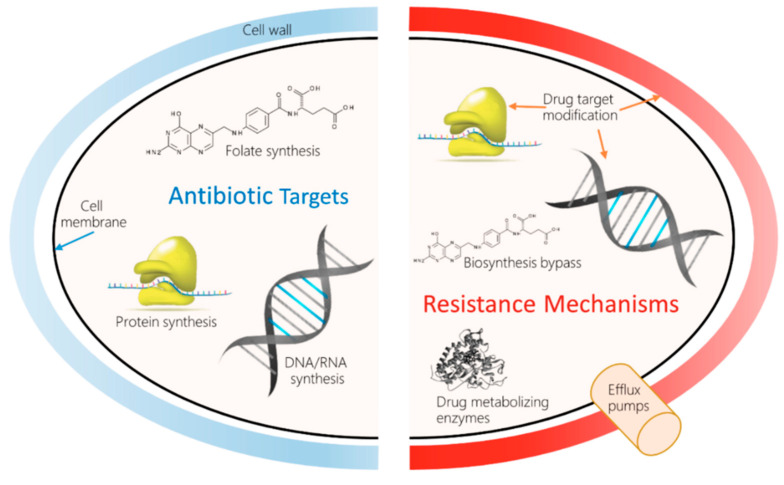
Molecular mechanisms of antimicrobial resistance (AMR) and drug resistance.

Antibiotics are a pillar of modern medicine. Their use has reduced childhood mortality. Beyond that, they are crucial for invasive surgery and complex treatments such as chemotherapy. They are irreplaceable aids in the complex era of robotics-guided surgeries, and for the management of secondary infections during sickness with the common flu, and infections of the skin, gut, etc. However, infections by multidrug-resistant bacteria are on the rise globally, causing the specter of untreatable infections to become a reality. A 2017 World Health Organization (WHO, Geneva, Switzerland) report confirmed that the entire world would will run out of antibiotics, as the existing drugs in clinical use were developed through modifications to the existing classes, and have shown to have short impact cycles [4]. The European Commission has documented the occurrence of AMR-related deaths to be around 33,000/year in the European Union (EU), costing about €1.5 billion/year in healthcare costs [5]. The 2019 antibiotic resistance threat report published by the Center for Disease Control and Prevention estimated the occurrence of AMR to be over 2.8 million/year in the US, including over 35,000 deaths. The report also further detailed threats under urgent, serious, concerning, and watch list categories; carbapenem resistance was considered urgent. The emergence of carbapenem-resistant bacteria raises a serious concern, as these are categorized as the “last-resort” antibiotics for treating multi-drug resistance infections. Reports on positive outcomes from collaborative research have documented the identification of novel resistance-busting antibiotic combinations to widen the usability of last-resort antibiotics [6]. Table 1 and Figure 1 summarize the different mechanisms underlying the development of antibiotic resistance.

## 2. Development and Identification New Antibiotics and Repurposing of Existing Antibiotics

Monitoring the use of available antibiotics has been popularly recommended to tackle rising incidences of antibiotic resistance, apart from the pressing need for identifying new target molecules and redesigning of the existent one to tackle the mounting adversity from MDR and XDR pathogens. Different aspects that can be included in development include:

(i) developing an enhanced derivative from an existing antibiotic family;
(ii) designing structurally superior anti-bacterial agents (new chemical structures);
(iii) identifying the benefits of alternative classes of agents, including phages.

One of the key strands of red-tape is regulatory aspects around certification, which adds to the complexity in the pipeline of developing new antibiotics, making it less lucrative for pharmaceutical companies to invest time and effort. The current situation indicates that urgently adopting new ways to fight antibacterial resistance is necessary. One such widely discussed approach includes the principle of nanotechnology, and the use of “nano functionalization” to re-establish the antimicrobial activity of existing antibiotics. A report by Natan, M. and Banin, E. (2017) details the use of nanotechnology to combat MDR, wherein nanoparticles (NPs) in two functional categories were introduced [7]. These two categories include NPs with intrinsic antibacterial properties and those which deliver antibacterial agents. In the latter case, the antibacterial action occurs through direct contact with the pathogen in question, hence making some antimicrobial resistance mechanisms void. A report by van der Meij, A. et al., (2017) focused on antibiotic production by actinomycetes [8]. These filamentous bacteria have been documented to survive in symbiosis with other organisms and offer protection and growth promotion. Biosynthetic gene clusters have been suggested to be expressed in response to host demand due to different environmental triggers. This insight has been documented to be beneficial in drug discovery.

Tracanna, V. et al. (2017), in a report discussing the diversity of functions of prokaryotic antimicrobial compounds, highlighted different mining strategies, including ribosomal synthesis and post translational modification of peptides, polyketides, etc [9]. These strategies are key to mining for unknown and novel biosynthetic gene clusters to narrow down on potential antimicrobial agents. Although this process is laborious, it indicates the need to combine genome information with functional and ecological data. To ensure optimal usage of the abundant sequenced genomes, new tools have been developed that can identify and repurpose existing and novel antimicrobial molecules. Presently, thousands of gene clusters have been discovered with the potential to encode for promising biomolecules. Ecological information aids in prioritizing key antimicrobial functions. Further, a function-based mining approach based on predicted function includes the use of information on protein domains for genome mining, or the use of information on mode of action. A report by Pachon-Ibanez, M.E. et al. (2017) addresses the possibility of defensins as antimicrobial therapeutic agents [10]. This review highlights advances in the development of human defensins and cathelicidin LL-37, along with their derivatives, as potential clinical antimicrobial agents. Apart from discussing the modes of action of the agents and their analogues, the study also highlights host-mediated defensive actions, and the three key factors that prevent the widespread use of promising related candidates: stability, toxicity, and cost [10]. Research over the years has highlighted the inherent struggles around antimicrobial agents and the MDR threat, even as human science continues to evolve our understanding beyond the cellular level. However, to achieve clinical success, it is essential for research efforts and funding allocated for basic and applied research to be significantly increased.

## 3. Factors Contributing to Antibiotic Resistance

**Figure 2:**
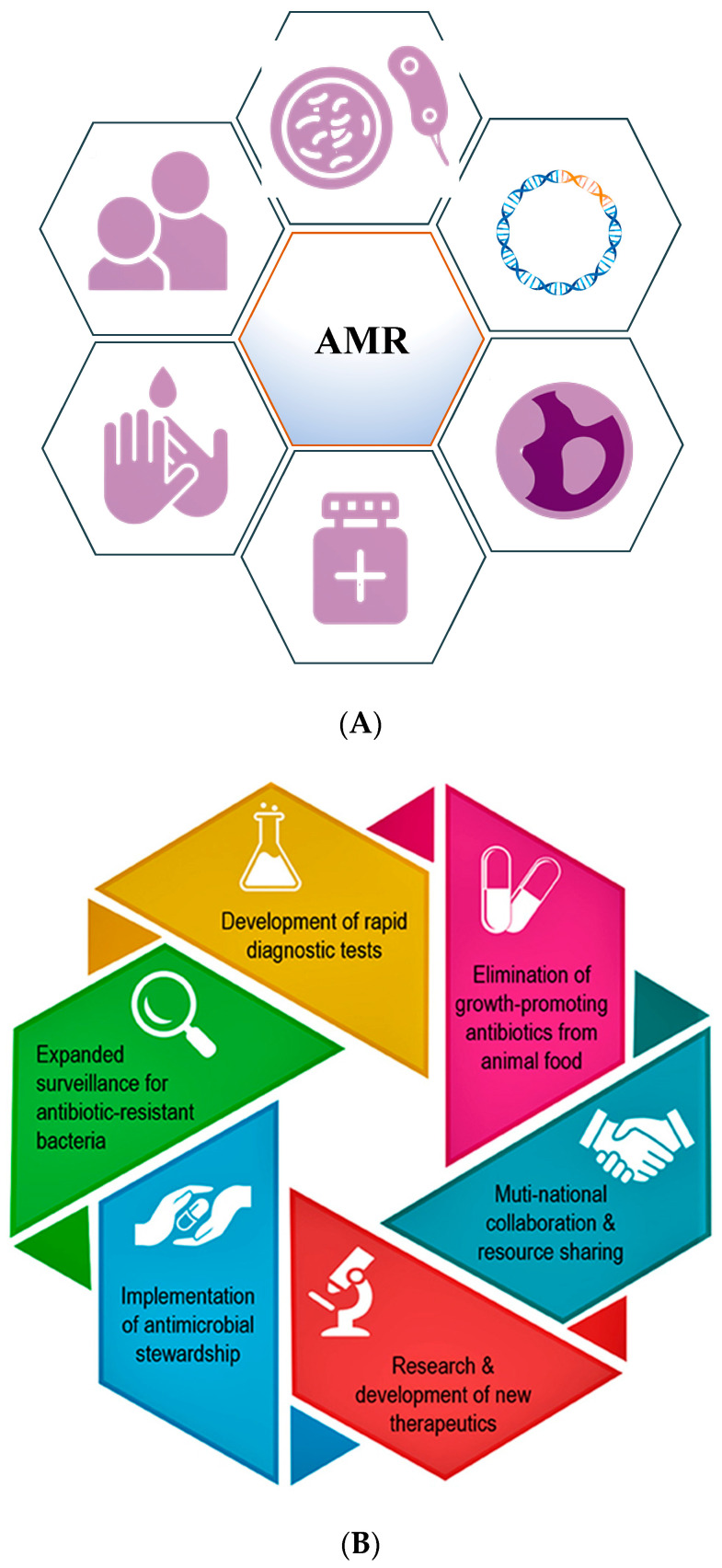
(**A**) Diagram highlighting the main steps that need to be implemented to minimize antimicrobial resistance. (**B**) Schematic representation of key aspects that can positively impact the global fight against AMR (modified from Dr. Razzaque’s paper).

Throughout history, the development of antibiotic resistance has been documented to be a natural process to enable survival of the bacteria, but has been fueled by human activities, including over-prescription and inappropriate prescription, overuse, excessive use of antibiotics as growth supplements in livestock, and the availability of few new antibiotics [11]. Numerous recent publications have underlined the consequences of promoting antimicrobial stewardship among medical professionals to lower complications around antibiotic use. These programs empower the medical community even at the undergraduate level with the knowledge to handle the antibiotic resistance crisis, a critical aspect of public health [12,13]. Figure 2A,B (modified from Dr. Razzaque’s paper) highlights the fundamental steps and key aspects involved in positively impacting the measures taken against AMR.

- Antibiotic resistance occurs when the target microbe develops a physiological mechanism to overcome the drug’s impact. This may be due to a change in bacterial envelope structure or composition, production of enzymes that break down the target agent, etc. Other mechanisms include limiting drug influx and increasing efflux, or modification/inactivation of drug target [14]. Apart from the natural process around the development of resistance, present-day usage practices around improper prescription, including that of broad-spectrum drugs, adds to the AMR crisis [15].
- AMR, apart from adding to the challenge around disease management, also impacts the patient. It has been documented to compromise the human immune system, and increase complications and vulnerability after complicated surgeries involving cancer, knee replacement, dialysis, etc. Further, individuals with comorbid conditions have an increased risk of severe adverse outcomes with AMR. Conditions which necessitate the use of “last-resort” antibiotics also significantly increase treatment costs to the client, prolonging in-hospital time and admission rates [16,17].
- The mounting evidence around antibiotic usage practice being a crucial risk towards AMR necessitates the need for inculcating habitual and appropriately guided clinical management practices. Knowledge around vaccinations, transmission, and prevention strategies are the key in public health education. Elaborate care practices for wounds and infections among patients with comorbid conditions can reduce the burden on hospital admission, and control infection spread.
- Communicating the need for antibiotics based on diagnosis and recommended clinical management protocol is also a crucial aspect in AMR. A lack of diagnostic tools and regulatory guidelines, and self-treatment with over-the-counter antibiotics for ailments such as the common cold and flu are common in developing countries, adding severely to the burden of AMR [18]. Further, reducing or controlling financial incentivization around prescription of antibiotics for physicians through pharmaceutical companies needs to be undertaken to avoid antibiotic usage abuse [19].
- Apart from changes in antibacterial use and consumption patterns across different global economies, modern day travelling has also been a major contributor towards dissemination of new infections and antibiotic resistance across the world. The recent coronavirus disease (COVID-19) pandemic is the best example. One documented study among European travelers from India identified the presence of carbapenemase-producing Enterobacteriaceae (CPE), even among those with no contact with the Indian healthcare system during their stay [20,21].

## 4. Factors Contributing to AMR Transmission

Transmission of highly drug resistant microbes has been documented to occur inter- and intra-species. Identifying the reservoirs and best practices around causes for transmission will be fundamental to controlling future pandemics. Pandemics and epidemics involving COVID-19 are examples involving viruses which spread material between species. Any public healthcare measure that can control the dissemination and use of antibacterial agents is the first step in controlling AMR. High-quality global surveillance systems are needed to provide warnings associated with changes in antimicrobial use, and avoidance of the time lag in knowledge transmission is a key to preventing a global health crisis by AMR.

## 5. Actions to Fight Antibiotic Resistance

Antibiotic resistance development is a gradual process wherein the microbes in question develop resilience against agents targeted towards their destruction. Addressing this threat needs aggressive action towards:

- Preventing infections and controlling transmission.
- Improving antibiotic use to slow the development of resistance through high quality surveillance and usage guidelines.
- Stopping the spread of resistant microbes when they do develop through antimicrobial stewardship programs.

## 6. Microbiome-Antibiotic Interactions

The science of the gut microbiome has presented ever-expanding opportunities to study the human—microbiome interaction, and provide an ecosystem to assess microbiome—antibiotic interactions. The impact of antibiotics on the human gut’s microbiome continues to be a key topic. Antibiotic exposure in early childhood has been linked to immunological, neurological, and gastrointestinal impacts; and antibiotic use at any age has been linked to negative impacts on the gut microflora leading to the emergence of antibiotic-resistant diarrhea [22]. Apart from AMR, studies have also implicated impacts of antibiotics on diseases, including obesity, cancer, and inflammatory bowel disease [23]. This reiterates the need for controlling the use of antibiotics even regardless of the AMR crisis. To restrict the effect of antibiotics on gut microbiota, establishing an antimicrobial stewardship program and curing dysbiosis through prebiotics, probiotics, and fecal microbiota transplantation have been suggested to be beneficial [24]. The factors around the rise in antibiotic resistance genes (ARGs) are also possible to evaluate through the gut microbiota. Studies have documented gut microbiota to be a rich reserve of antibiotic resistance (AR) which contributes to the emergence of multidrug resistance by horizontal gene transfer (HGT), which is also triggered during use of antibiotics as vital prophylaxis [25].

Antibiotic-induced microbiome depletion is a well-documented phenomenon due to antibiotic therapy and has been implicated in the alteration of metabolic homeostasis through gut signaling [26]. Studies have also documented gut microflora metabolites to be key to the gut—brain axis, including short-chain fatty acids, bile acid metabolites, etc. Changes in the microbial-metabolic pathways have been linked to Alzheimer’s disease, anxiety, depression, etc. [27].

Studies have also documented the impact of administration of broad-spectrum antibiotics to healthy people after seasonal influenza vaccination on the gut microflora. Analysis found enhanced inflammatory signatures and a reduction in bile acids causing inflammasome activation [28]. Autologous fecal microbiota transplantation (a-FMT) has been widely studied as an intervention to treat dysbiosis caused by infectious disease such as *Clostridioides difficile* infection (CDI), with which cure rates of up to 90% have been recorded. Other conditions include inflammatory bowel disease, with the respective clinical efficacy ranging between 25 and 50%. The differences in the impact of FMT have also been attributed to differences in microbiome composition between donors and recipients, due to changes in diet and environment at different time points [29,30].

**Figure 3:**
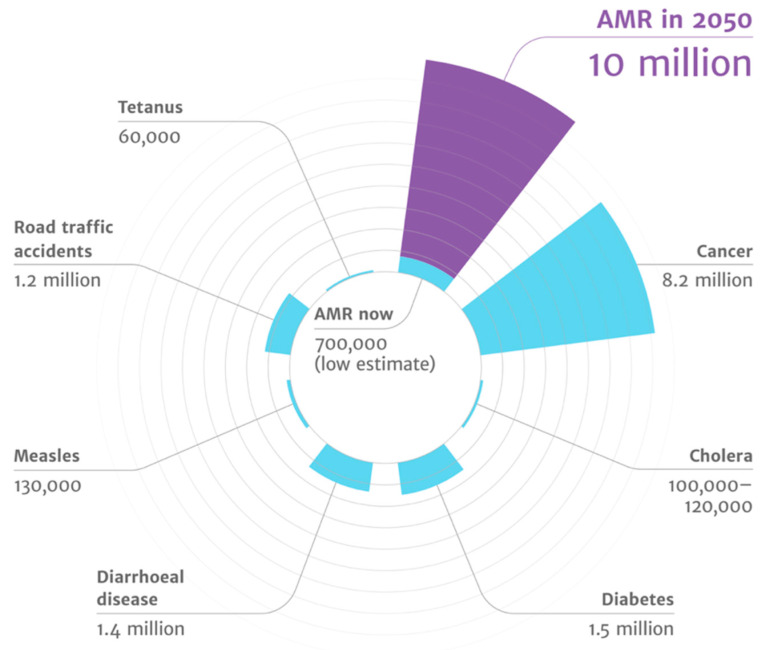
AMR has highest disease burden (source: the review on antimicrobial resistance chaired by Jim O’Neill).

The majority of the orally administered drugs experience exposure to the gut commensals of the host, and the human microflora has been shown to respond through the drug-metabolizing enzymes. The gut microflora has been shown to act in synergy with the host to maintain intestinal homeostasis, metabolizing xenobiotics and drugs by altering expression levels of drug transport receptors and drug-metabolizing enzymes [31]. The gut microflora has also been shown to impact the bioavailability of phytochemicals, including metabolic intermediates such as glycosidases and demethylations [32]. Further, metabolism of curcumin has been associated with *Escherichia coli* [33]. The gut microbiota is also involved in activation and deactivation of drugs such as sulfalazine, digoxin, irinotecan, chloramphenicol, and nitrobenzodiazepine, to name a few [34,35,36,37]. A recent study evaluated the impact of seventy-six diverse human gut bacteria on the metabolism of two-hundred and seventy-one oral drugs using high-throughput sequencing with mass spectrometry. This study found 176 drugs to be significantly metabolized by a minimum of one bacterial strain, and each strain metabolized between 11 and 95 different drugs [38]. Such advances highlight the possibility of designing personal medicines which can act in conjunction with the existing practices of pharmacogenomics. Figure 3 highlights that antimicrobial resistance leads to the highest disease burden, as highlighted in the review on antimicrobial resistance by Jim O’Neill [39].

## 7. Applications of Technology against Antibiotic Resistance

Studies have described the antibiotic resistome to be dynamic, implicating the significance of cutting-edge technology to explore and study microbial diversity in-depth [40]. The emerging platforms such as next-generation sequencing (NGS) technology, bioinformatics tools, and large-scale dynamic public databases, have accelerated research elucidating antibiotic resistance. Technological advancements integrate deep mechanistic understanding of AMR with high-depth genomic analysis, aiding in improvement of surveillance, and developing proactive strategies to mitigate threats [41].

Metagenomics is the currently most-widely used approach to decipher the resistome. Three methods, including shotgun metagenomics, functional metagenomics, and targeted gene sequencing, have been adopted and advocated by several studies over the last decade. Studies have characterized the pediatric gut-associated resistome using metagenomic recombinant libraries, leading to identification of resistance towards 14 of the 18 antibiotics tested from eight drug classes. Further, few of the recovered genes included drug-resistant dihydrofolate reductases, chloramphenicol acetyltransferases, multidrug efflux pumps, and every major class of beta-lactamase. Many resistance-conferring sequences were also found to be mobilizable [42]. Functional metagenomics with shotgun-cloned deoxyribose nucleic acid (DNA) fragments have been increasingly used towards identification and characterization of antibiotic resistance reservoirs [43]. These studies highlight the significance of using high-performance sequencing platforms, appropriate ARG databases, and analysis pipelines to identify AMR at the resistome level. The SmartChip system (Takara, Shiga, Japan), which is a high-throughput quantitative polymerase chain reaction array, has also been widely used for resistome studies involving various environmental microbiomes [44,45,46]. This system has a number of advantages, including the ability to analyze a high number of ARGs in parallel within a short time, and has higher sensitivity than the metagenomics approach for detecting ARGs. For resistome analysis, whole-genome sequencing for identification of antimicrobial-resistance determinants is preferred due to technical feasibility, cost efficacy, and the ability to identify actionable results with reference to infection control [47].

## 8. Antimicrobial Resistance Surveillance through Next Generation Sequencing (NGS)

The concern around the increasing incidence of multi-drug resistance cases is on the rise, and hence, surveillance and understanding of ARGs are keys in monitoring their emergence and spread. ARGs can be identified by different platforms, including:

(1) Microarray [48,49].
(2) Polymerase chain reaction (PCR) [50].
(3) Whole-genome sequencing (WGS) [51].
(4) Metagenomics [52].

## 9. Whole Genome Sequencing Approaches for Surveillance of Resistance

Advancements in genomics towards detection of resistance to antibiotics has not seen successful implementation in clinical laboratories, wherein traditional serial dilution and diffusion methods are still in use [53]. These techniques, though, have been promising in the design of anti-infective therapy. For ARG surveillance, traditional techniques have several disadvantages. A few include the lack of a validated and sensitive approach for several microbes, inter-laboratory conditions, and a limitation in the number of drugs tested. Surveillance of ARG is also ineffective without including a comparison of genotypes across multiple hosts and environmental zones, enabling route tracking and the degree of spread [54,55].

The genetic signature determined through WGS displays high precision information, though which vital microbial identification, establishing phylogenetic relationships, mutation detection, enhancing the identification of putative new genes, and predicting phenotypic antibiotic susceptibility become possible. Genome analysis can also provide valuable insights on antibiotic resistance traits. This is precious in the investigation of outbreaks and real-time surveillance and dissemination of ARG [53]. Surveillance of ARGs in real time aids in early identification of outbreaks and in the upgradation of public health policies. A study from the Gulf Health Council reiterates the need for WGS-based surveillance of AMR to identify burden and prevalence [56]. WGS has been the technology of choice under many circumstances involving tracing the sources of outbreaks and forming policies in recalling contaminated food. WGS also provides several other advantages over traditional resistance testing platforms, including finding the existence of co-resistance to other antibiotic agents, such as heavy meals; and predictions around identification of horizontal gene transfer [53]. Although WGS is a powerful tool for surveillance of ARG, requirements around culture purity standards add to the technical complexity. Further, the absence of the “culturability” aspect of most bacterial and archaeal taxa stands is proven [57].

ARGs surveillance is an essential part of the Global Antimicrobial Resistance Surveillance System launched by the WHO is the year 2015 as the first collaborative effort towards standardizing AMR surveillance. It provides a structural framework to collect, analyze, and share AMR data; and documents the status of existing and new AMR surveillance systems. It encourages a shift from an isolate-based data system to epidemiological and clinical-level data. It aims to generate standardized data at standards that are comparable between nations, serve as a guideline in forming policies, track the spread and emergence of ARGs, and guide resource allocation to handle the ARG threats [58,59]. The integrated surveillance system of antimicrobial resistance has tailored to the ever-changing requirements and threats through expanded surveillance of catchment areas, studying new isolate source, devising sampling schemes, and modifying the tested antimicrobial agents [55]. The Initiative by the European Centre for Disease Prevention and Control, in the EU, has been in existence for investigation and tracking of outbreaks [5]. These reiterate the need for the establishment of similar monitoring bodies the world over, including the GHC for surveillance of ARGs, and tracking and investigation of outbreaks in Saudi Arabia. Several measures have been proposed to address the challenges posed by resistance to antibiotics, and to overcome this crisis, including public education, and policy measures which are effective when in dynamic motion, but reducing the inessential usage of antibiotics at the international level is still the key [47].

A review on antimicrobial resistance from 2016 highlights that inaction towards AMR could lead to a loss in global productivity to the tune of 100 trillion USD by year 2050. Further, few government initiatives in the western world have been funding research in this regard, one being the Fleming Fund by the UK government initiated to improve surveillance on drug-resistant infections in the low- and middle-income countries. The report also states the disparity in investment in antimicrobial research, which was only 1.8 billion USD of the 38 billion USD invested (<5%) by venture capital funds in R&D between 2003 and 2013 [60].

## 10. Metagenomic Approaches for Resistance Surveillance

**Figure 4:**
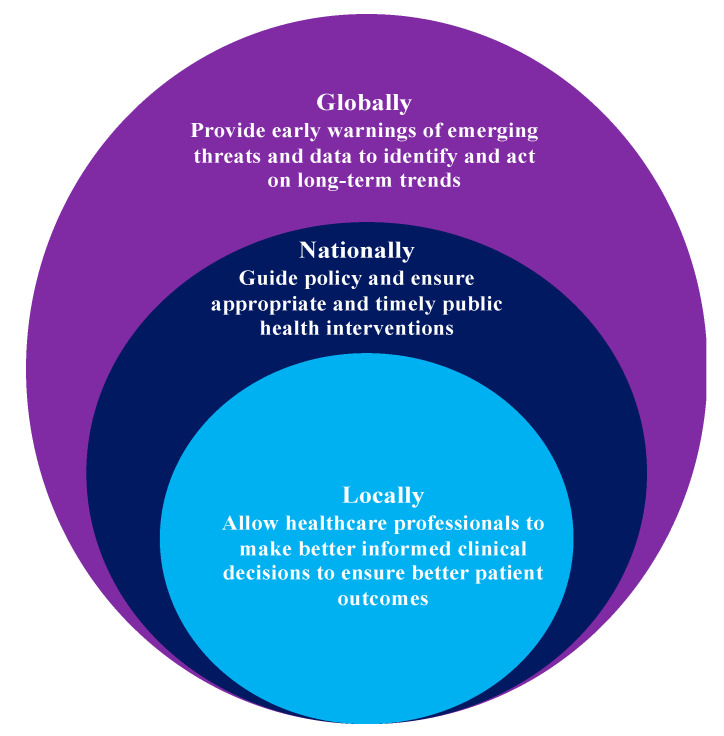
How surveillance can improve health outcomes.

Metagenomics is documented to be the best technological advancement towards identifying the entire spectrum of AMR from various sources, including livestock and human gut microflora. Descriptive metagenomics plays a key role in identifying microbiome community structure and variations in microbial abundance under different environmental and physiological conditions. Functional metagenomics studies host–microbe and microbe–microbe interactions in a predictive dynamic ecosystem [61,62]. Metagenomics has been documented to be a monumental next-generation addition to molecular taxonomy, as it established a standard typing method which overcomes the obstacles imposed by standard-culture methods; and it is purely sequence- and function-driven [63]. Discovery of novel mechanisms around AMR and identification of ARG reservoirs have been attributed to structural metagenomics analysis. The solutions to AMR regarding discovery of novel antibiotics, antibacterial proteins, and antibiotic synthesis pathways have been promised by functional metagenomics [64]. Figure 4 highlights how surveillance can improve health outcomes as per WHO.

### 10.1. Identification of AMR Genomic Signatures from WGS Data

The fundamental approach to identifying the presence of ARGs involves studying the whole genome sequencing data, for both the endogenous elements and plasmids. The data on presence/absence and abundance of vectors have been used for the development of machine learning models to predict the occurrence of AMR in samples. A report on prediction of AMR by WGS in *Staphylococcus aureus* involved the assembly of genomes by the BLASTn algorithm [65]. Another report on a WGS-based web tool termed PointFinder identified point mutations in bacterial pathogens that are causative for AMR through a database, and studies using BLASTn to identify genes in the database and hits with identity >/= 80% were further analyzed [66]. PhyResSE has been used for delineating *Mycobacterium tuberculosis* antibiotic resistance and lineage through WGS data. It consists of validated resistance data from well characterized samples from the world over, and is tailored for MTBC antibiotic resistance diagnosis [67]. The tool applies BWA-MEM to map isolates with references more rapidly than the BLASTn sequence aligner to identify isolate lineage from a pre-compiled catalog of 92 lineages with known resistance phenotypes [68]. The “Mykrobe predictor” employs an alignment-free approach through de Bruijn graphs to build a library of reference strains of *M. tuberculosis* and *S. aureus* resistant and susceptible to multiple antibiotics. It can identify species, build resistance profiles, and analyze other aspects, such as phylogenetic lineages and virulence elements. The impact of extended research using WGS data led to the publication of the CRyPTIC consortium report of over 10,000 clinical isolates of *M. tuberculosis* from 16 countries representing all major lineages, and concluded WGS to be efficient for characterizing profiles of susceptibility to first-line antituberculosis agents, with sufficient accuracy for clinical use [The CRyPTIC Consortium and the 100,000 Genomes Project]. Though the panel is often predefined for a set of ARGs, the high confidence prediction makes it suitable for clinical use.

### 10.2. AMR Gene Signatures from Expression Data

Another approach to AMR prediction is through differences in gene expression of the isolate post exposure to the target drug. A report by Suzuki S et al. [69], details genotypic and phenotypic changes in antibiotic-resistant bacterial strains, such as *E. coli*. This study found acquisition of resistance to one drug changes susceptibility and resistance to others. Subsequent gene expression analysis led to the identification of dynamic compensatory mechanisms. Teixobactin, a new antimicrobial agent has documented activity against *S. aureus* and *Enterococcus faecalis* without resistance. A study which reported on the transcriptional response of *E. faecalis* to teixobactin levels identified development of intrinsic tolerance to high concentrations of the same (through deletion of *croRS*), and this is generally considered a precursor for the development of resistance [70]. Besides known mechanisms, involving differential expression of genes targeted by antibiotics, and xenobiotic efflux transporters, emerging studies have the potential to reveal regulatory circuits that respond to different toxic stimuli and other compensatory mechanisms. The major setbacks in clinical implementations, however, include experimental complexity and the time spent to analyze and interpret the data. Moreover, the lack of annotation of all the clinically important pathogens (and their strains), such as *E. coli*, and identification of differentially expressed transcripts, are further delayed by the need for functional annotation to decipher their role in AMR.

### 10.3. Identification of AMR Mechanisms Agnostic to ARG

This approach elucidates AMR mechanisms based on global genomic comparisons with various susceptibility profiles of multiple strains. Another proof-of-concept study recorded the significance of stability selection to investigating the bacterial genotype–phenotype relationship in *M. tuberculosis* and *S. aureus* as a powerful approach. This study highlighted the significance of genotyping with *k*-mers, and the benefits include being alignment-free and the ability to identify different categories of genetic variants, including single nucleotide insertions and deletions in the coding and non-coding regions. The study identified *k*-mers (*n* = 1 to 8 per antibody; 22 total), which were further used in linear regression models for each tested antibody and found to have performance on par with Mykrobe [71].

Studies have highlighted the significance of machine learning with pan-genome structural analysis of *M. Tuberculosis* to identify genetic signatures of antibiotic resistance. Subsequent mapping to available 3D structures of select proteins associated with AMR allowed the authors to pinpoint mutation clusters of certain proteins in the antibiotic response. This insight may help to better explain the AMR causality of alleles [72]. Another recent report explored the performances of two learning-based models, classification and regression trees, in AMR prediction. The study found they were highly accurate models, and the interpretation revealed known resistance mechanisms for 12 species and 56 antibiotics, and potentially new ones [73].

The pan-genome approach is funding intensive due to need for genomic sequencing technology and tools for a genome-wide comparison approach, although it holds great promise for clinical applications. An advantage is its ability to explore entire genomes instead of known AMR-related regions, to identify new mechanisms or regulatory elements. This is crucial to determining if the relation between a genomic signature and AMR is a statistical probability or causal. Furthermore, the need for large collections of strains is key to obtaining informative insights into AMR, which adds to the challenge.

### 10.4. Deciphering AMR Mechanism from Metabolomics Data

Studying metabolic profiling has also been widely studied as an approach orthogonal to the ones discussed previously. A report by Yang JH et al. [74] discussed the integrated “white-box” biochemical screening network modelling and machine learning approach to identify causal mechanisms and to apply the same in understanding antibiotic efficacy. The study counter-screened three antibiotics (ampicillin, ciprofloxacin, and gentamicin) against *E. coli* and simulated the corresponding metabolic status via genome-scale metabolic network model. The white-box machine learning approach yielded information on pathway mechanisms, facilitating the quantification of the relative contributions of each metabolic pathway towards tackling lethal mechanisms of each antibiotic. Further, profiling of over 500 intracellular and extracellular putative metabolites involving the 190 evolved populations highlighted carbon and energy metabolism to constrain the speed and mode of resistance acquisition. The study further identified multiple mechanisms employed by the *E. coli* strain to compensate for the pressure from different antibiotics, including mutations or expression of efflux pumps, which was further found to be influenced by the carbon source, and therefore, the metabolic state the organism is in [75]. This approach highlights an evolved perspective on the understanding of antibiotic action and response of bacteria to drugs.

## 11. Conclusions

Resistance towards antibiotics is an inevitable part of microbial evolution. This emphasizes the need for surveillance of ARGs as the key aspect in future policy making. WGS and metagenomics, coupled with powerful bioinformatics tools and databases, have only furthered our knowledge in this regard. The reliability of results from these techniques foster encouragement for their execution in routine diagnostics. Use of antibiotics leads to dysbiosis of the gut microbiome, causing several side effects. In the monitoring of AMR in general and in the implementation of NGS technologies in particular, each domain has its own set of technical challenges, requirements, and realities. The only way forward is continuous education, surveillance, data analysis, and research to tackle the ever-emerging AMR crisis. A global public awareness campaign tackling the supply problem, control of antibiotic usage, and stimulus to the diagnostic market to initiate more rapid diagnostic tests, has been discussed as a key intervention with which to tackle AMR.

The bioinformatics approach to AMR identification includes the aspects that focus on reliable insights of AMR that are replicable in clinical settings. This is focused towards underlining new genes of resistance, the regulatory mechanisms, or new antibiotic targets. Every approach has limitations which should be taken into consideration when building and training superior prediction models. The combination of advanced molecular technologies and powerful artificial intelligence and machine learning algorithms holds great promise for understanding AMR at the molecular level to generate clinically relevant predictions, leading towards selection of personalized antibiotics geared for favorable clinical outcomes.

## Figures and Tables

**Table 1:** The mechanisms of antibiotic resistance.

Mechanism of Resistance	Antibiotic Type	Example
Hydrolysis, efflux, altered target	P-Lactams	Penicillins, Cephalosporins, Penems, Monobactams
Phosphorylation, acetylation, nucleotidylation, efflux, altered target	Aminoglycosides	Gentamicin, Streptomycin, Spectinomycin
Reprogramming peptidoglycan biosynthesis	Glycopeptides	Vancomycin, Teicoplanin
Monooxygenation, altered target, efflux	Tetracyclines	Minocycline, Tigecycline
Hydrolysis, efflux, altered target, glycosylation, phosphorylation,	Macrolides	Erythromycin, azithromycin
Nucleotidylation, efflux, altered target	Lincosamides	Clindamycin
Carbon-Oxygen lyase, efflux, altered target, acetylation,	Streptogramins	Synercid
Efflux, altered target	Oxazolidinones	Linezolid
Acetylation, altered target, efflux,	Phenicols	Chloramphenicol
Acetylation, altered target, efflux,	Quinolones	Ciprofloxacin
Efflux, altered target	Pyrimidines	Trimethoprim
Efflux, altered target	Sulfonamides	Sulfamethoxazole
ADP-ribosylation, altered target, efflux,	Rifamycins	Rifampin
Altered target	Lipopeptides	Daptomycin
Altered target, efflux	Cationic peptides	Colistin
